# How much the leg length has changed after the MOUKA through measurement of the full length radiographs? Beware of splicing error

**DOI:** 10.1186/s12891-023-06472-0

**Published:** 2023-05-03

**Authors:** Tianlang Xie, Xufeng Jiao, Xiaomei Yao, Zheng Li, Shuai An, Guanglei Cao

**Affiliations:** 1 Department of Orthopedics, Xuanwu Hospital Capital Medical University, 45 Changchun Street, Xicheng District, Beijing, 100053 China; 2 Department of Health Research Methods Evidence and Impact, McMaster University, Hamilton, ON Canada; 3 Center for Clinical Practice Guideline Conduction and Evaluation, Children’s Hospital of Fudan University, Shanghai, China

**Keywords:** Unicompartmental knee arthroplasty, Leg length change, Full-length radiograph, Splicing error

## Abstract

**Background:**

Leg length change after knee arthroplasty is one of the most concerned problems for patients and doctors. However, as there was only one literture focused on the leg length change after unicompartmental knee arthroplasty, we aimed to clarify the leg length change after medial mobile-bearing unicompartmental knee arthroplasty (MOUKA) using a novel double calibration method.

**Methods:**

We enrolled patients who underwent MOUKA and had taken full-length radiographs in a standing position prior to and at 3 months after the operation. We eliminated the magnification by a calibrator and corrected the longitudinal splicing error by measuring the femur and tibia lengths before and after operation. Perceived leg length change was collected 3 months after operation. Bearing thickness, preoperative joint line convergence angle, preoperative and postoperative varus angles, flexion contracture and Oxford knee score (OKS) were also collected.

**Results:**

From June 2021 to February 2022, 87 patients were enrolled.76 (87.4%) of them showed an increase with an average of 0.32 cm (range from -0.30 cm to 1.05 cm) in leg length change. The lengthening was strongly correlated with the degree of varus deformity and its correction value (r = 0.81&0.92, *P* < 0.01). Only 4 (4.6%) patients perceived leg length lengthening after operation. There was no difference in OKS between the patients who had an increase in leg length and those who had a decrease (*P* = 0.99).

**Conclusions:**

Majority of patients only experienced a slight increase in leg length after MOUKA, and such an increase did not affect patients’ perception and short-term function.

## Background

The leg length change after knee arthroplasty has always been a focus of patients and doctors [1]. Some patients may suffer from leg length discrepancy caused by leg length change after operation, which can reduce the patient’s satisfaction with the operation. In addition, post-operative leg length discrepancy may result in many unwanted effects, such as poor recovery, limping, acceleration of contralateral knee osteoarthritis, and lower back pain [2–4]. As the total knee arthroplasty (TKA) is the most common operation for end-stage knee osteoarthritis, most of the research have been focused on the leg length change after TKA. A study suggested that 83% of the operations showed an increase in leg length with an average increase of 6.3 mm after TKA [5].

With the increase in popularity of stepwise treatment protocol of knee osteoarthritis, medial mobile-bearing unicompartmental knee arthroplasty (MOUKA) has been adopted by more and more orthopedist due to its advantages of decreased likelihood of trauma, preservation of patients ‘ ligaments and proprioception, quick recovery, and higher patient satisfaction rate [6, 7]. Unlike TKA, MOUKA aims to restore the normal tension of the medial collateral ligament through the replacement of the medial compartment, and preserves the physiological varus deformity of the patient [8]. Therefore, the leg length change after MOUKA may be different from that after TKA.

To our knowledge, there has only been one study by Zhao et al. that had reported leg length change after MOUKA [9]. The study concluded that among 114 patients, 78.9% of patients showed an increase in leg length with an average increase of 9.39 mm (from -21.00 to 33.79 mm) after MOUKA, based on the full-length radiograph taken ten days after the operation. Theoretically, MOUKA is a pure surface replacement, and the lengthening is only caused by slight angular changes in the coronal and sagittal plane of the lower extremity, so the length change should not be that great. For a more accurate result, we start our study.

The main purpose of our study was to test the hypotheses that the leg length change after MOUKA are very limited and most patients will not perceive such changes. Meanwhile, we supposed that: 1.the leg length change is related to the preoperative varus deformity and its improvement value; 2. the leg length change after MOUKA does not affect the short-term (defined as 3 months after MOUKA) function of patients.

## Methods

In this prospective study, patients were eligible if they: 1) underwent MOUKA because of end-stage disease of medial compartment of the knee; and 2) have taken full-length radiographs prior to and 3 months after the operation. They were excluded if they: 1) had previous hip operation; 2) had inclination of pelvis; 3) were diagnosed with lumber disease that affected the lower limb function; and 4) had the radiographs showed obvious rotation malalignment or transverse splicing error that might influence measurement.

All operations were performed by the same orthopedist. The medial parapatellar approach was used in all cases. We used Oxford medial unicompartmental implants with Microplasty Instrumentation (2 pegs, Zimmer Biomet, Warsaw, IN, USA) for prostheses. All patients followed the same rehabilitation protocol to minimize the flexion contracture and to increase the range of motion in the knee after operation.

We collected the following information: age, gender, height, weight, BMI, and bearing thickness. In addition, we used the full-length radiographs in the standing position to collect information regarding the leg length (LL), femur length (FL), tibia length (TL), hip-knee-ankle angle (HKA), the extent of varus deformity before and 3 months after the operation,, and joint line convergence angle (JLCA) before the operation. The flexion contracture was measured using a goniometer and the knee function was assessed using the OKS before and 3 months after operation. The difference between postoperative OKS and preoperative OKS was defined as △ OKS. In addition, we also recorded patients' perception of leg length change at 3 months after the operation.

**Fig. 1:**
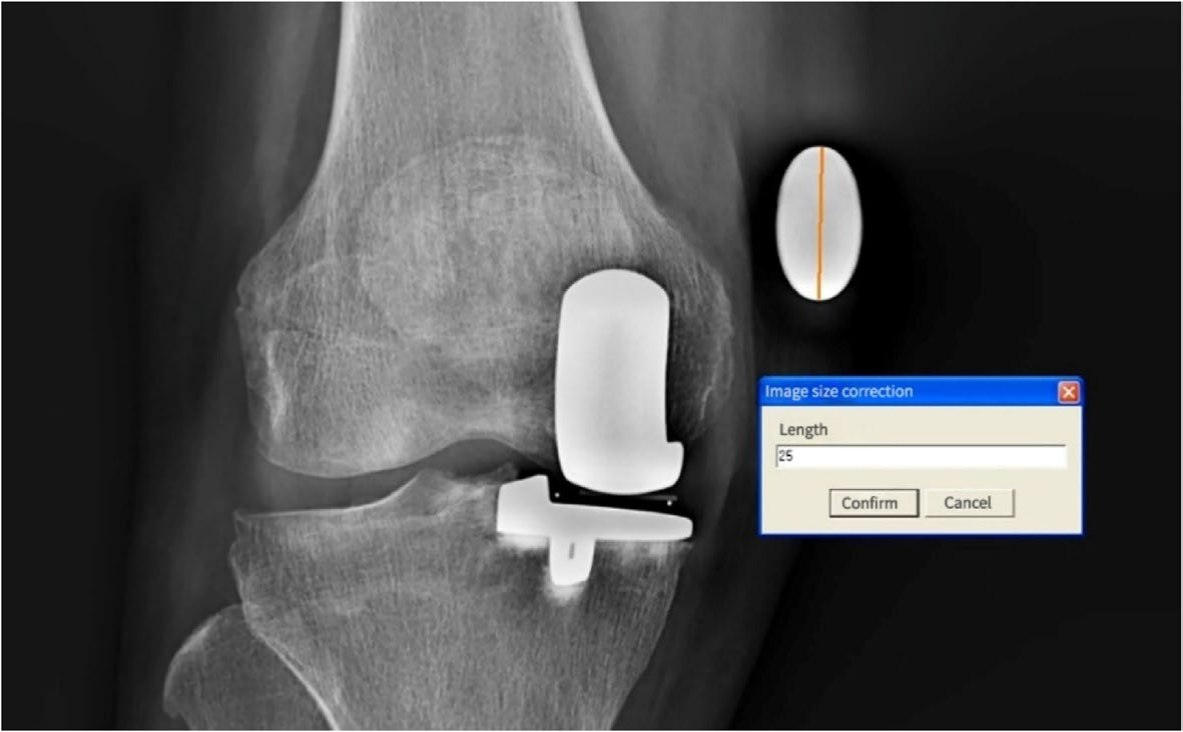
First calibration. To eliminate the any errors due to magnification, we used the diameter of the coin (25 mm) as reference when measuring

**Fig. 2:**
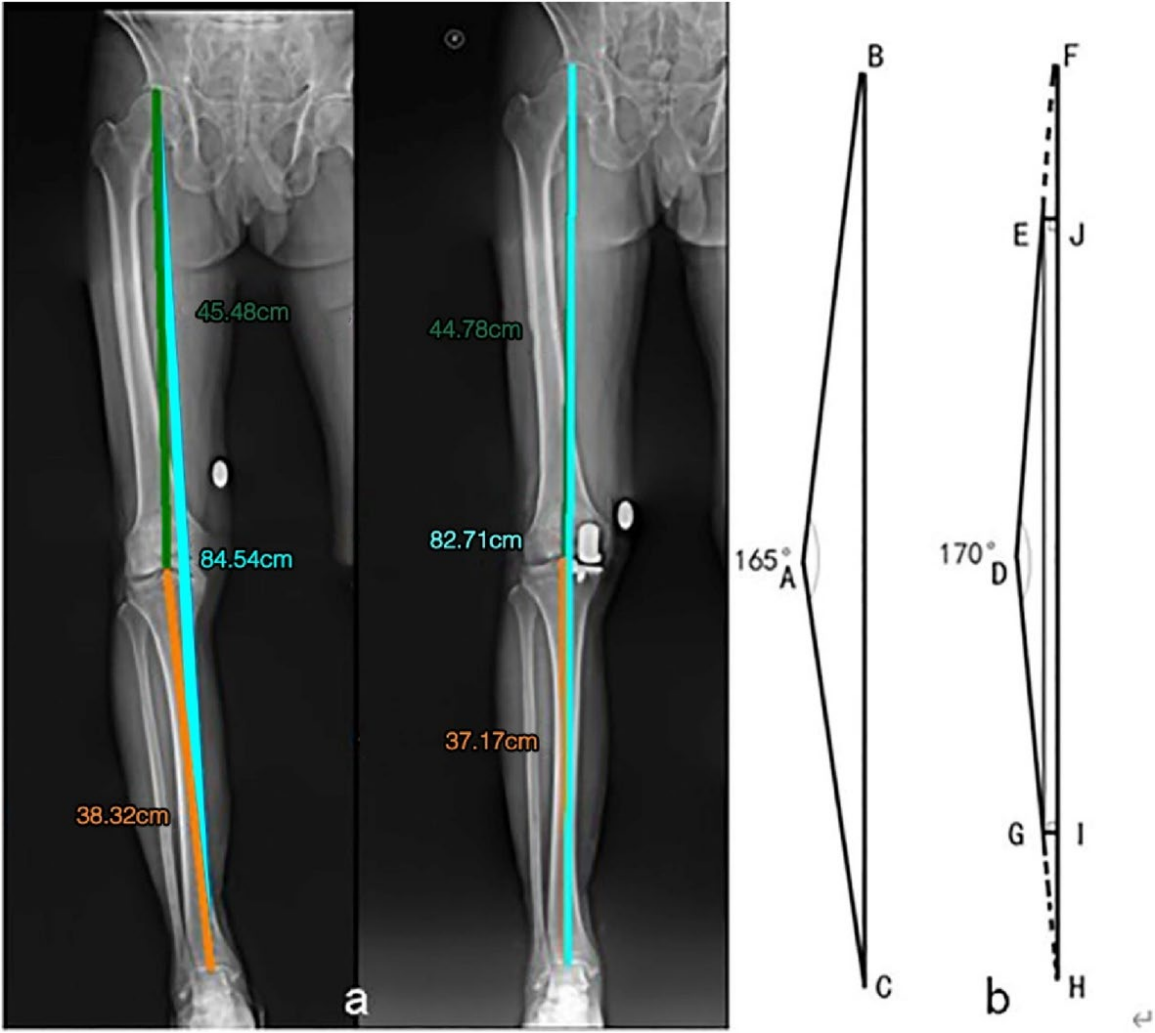
Legnth measurement and Second calibration. **a** Measurement of femur length, tibia length, leg length after first calibration. The leg length was measured from the highest point of the femoral head to the center of the distal tibia. The femur length was measured from the highest point of the femoral head to the middle of the femoral intercondylar fossa. The tibia length was measured from the middle of tibial plateau to the middle of articular surface of the distal tibia. **b** Second calibration: An example of underestimation of leg length due to splicing error. Line AB denotes the preoperative femur length. Line AC denotes the preoperative tibia length. Line BC denotes the preoperative leg length. Line DE denotes the post-operation femur length. Theoretically, line DE should be equal in length to line AB, but in actuality, line DE was shorter than line AB. We extended line DE to point F so that line DF is the same as line AB. Line EF denotes the longitudinal splicing error of the femoral end, and line JF was the "contribution value" of the change of leg length. The diagram applies to the tibial side as well. The real postoperative leg length (line FH) is line EG plus the lost length (lines FJ and IH) due to longitudinal splicing error

When performing the X-Ray examination, we asked the patients to extend their lower limb as straight as possible, with the patella pointed forward. In order to eliminate any magnification, a coin (25 mm in diameter) was attached to the distal medial side of the femur of each patient as a calibrator [10]. We used the Unisight (EBM Technologies Incorporated, Taipei, Taiwan, China) to measure LL, FL, and TL after calibration using the coin (Fig. 1). After the first calibration, we measured the LL, FL, TL respectively [11] (Fig. 2a). According to the principle that MOUKA does not affect the length of femur and tibia, we eliminated the longitudinal splicing errors through secondary calibration (Fig. 2b). We used MAYA (2018, Autodesk, California, USA) to build a physical model to simulate the leg length change after operation.

**Fig. 3:**
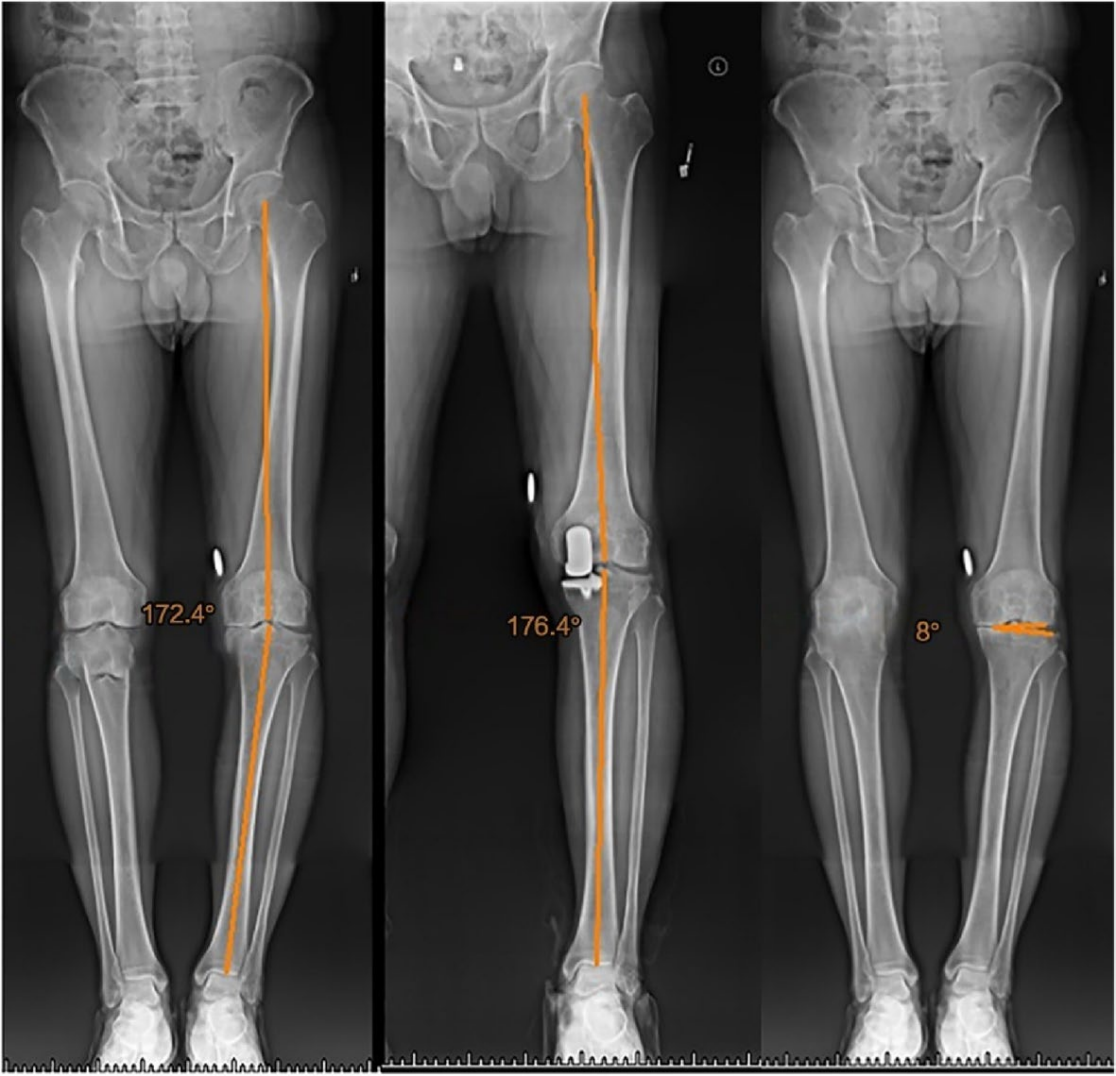
Preoperative and postoperative hip-knee-ankle angles and preoperative joint line convergence angle was measured in full-length radiograph

HKA is the inner angle between the mechanical axis of the femur and tibia, and the angle is considered to be varus if it is less than 180°. Its supplementary angle is the varus angle. Patients were divided into two groups based on their preoperative varus angle: varus angles ≥ 5° and < 5°. The difference between preoperative and postoperative varus angles was defined as △ varus angle and patients were divided into two groups, those with △ varus angle ≥ 5° and < 5°. The JLCA was measured as the angle between the tangent of the distal articular surface of the femur and proximal surface of the tibial plateau, and patients were divided into JLCA ≥ 5° and JLCA < 5° groups (Fig. 3).

**Fig. 4:**
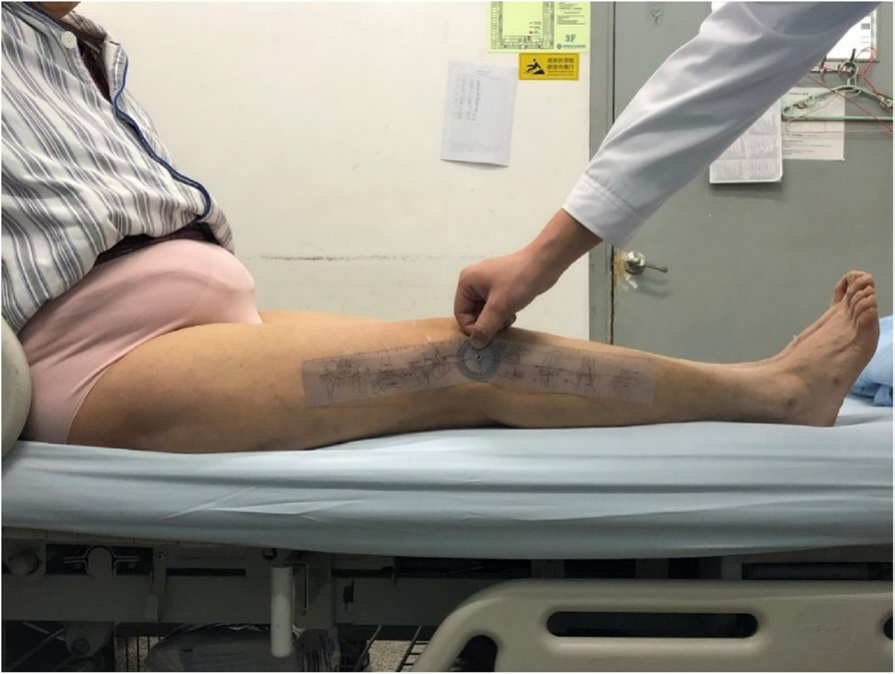
Measuring the flexion contracture angle with a goniometer

To measure the flexion contracture angle, the patient was asked to sit and extend the knee as straight as possible, and then the goniometer was used to align both ends of the lower limb with the axis of femur and tibia, respectively [12] (Fig. 4). The difference between the preoperative and postoperative flexion contracture angles was defined as △ FC.

Based on the preliminary test (Power = 90%, α = 0.05, mean = 0.50 cm, standard deviation = 1.40), we calculated the sample size by “Test for Paired means” in PASS (2017, NCSS, LLC. Kaysville, Utah, USA) and showed that we need to enroll 68 patients. Consider the 10% drop-out rates, we should include 75 patients at least.

The data analysis software used in this study was IBM SPSS Statistics 25 (IBM, Armonk, NY). Continuous variables were expressed as mean ± standard deviation. The comparison of lower limb length between different groups before and after surgery was analyzed using paired T-test and independent-sample T test. Pearson or Spearman correlation analysis was applied to the correlation between continuous variables. *P* < 0.05 was considered significant.

## Results

A total of 127 patients underwent the operation from June 2021 to February 2022. Based on the inclusion and exclusion criteria, a total of 87 patients (87 knees) were included in this study eventually (Table 1).

**Table 1:** Demographic data (BMI: body mass index)

Parameters	Total	Varus ≥ 5°	Varus < 5°	△varus ≥ 5°	△varus < 5°	JLCA ≥ 5°	JLCA < 5°
**N**	87	66	21	38	49	40	47
**Age**	68.2(10.4)	69.0(7.6)	65.8(16.4)	69.1(7.6)	67.6(12.1)	68.4(8.6)	68.1(11.7)
**BMI (Kg/m**^**2**^**)**	27.0 (3.3)	27.2(3.3)	26.5(6.5)	27.0(3.3)	27.1(3.3)	27.6(3.0)	26.5(3.5)
**Side(L/R)**	46/41	34/32	12/9	22/16	24/25	20/20	26/21
**Gender (Male/Female)**	19/68	15/51	4/17	6/32	13/36	8/32	11/36
**Bearing thickness (3/4/5) (mm)**	68/17/2	50/15/1	18/2/1	29/8/1	39/9/1	26/13/1	42/4/1
**Number of patients who perceived leg length lengthening (N)**	4	4	0	4	0	4	0

**Fig. 5:**
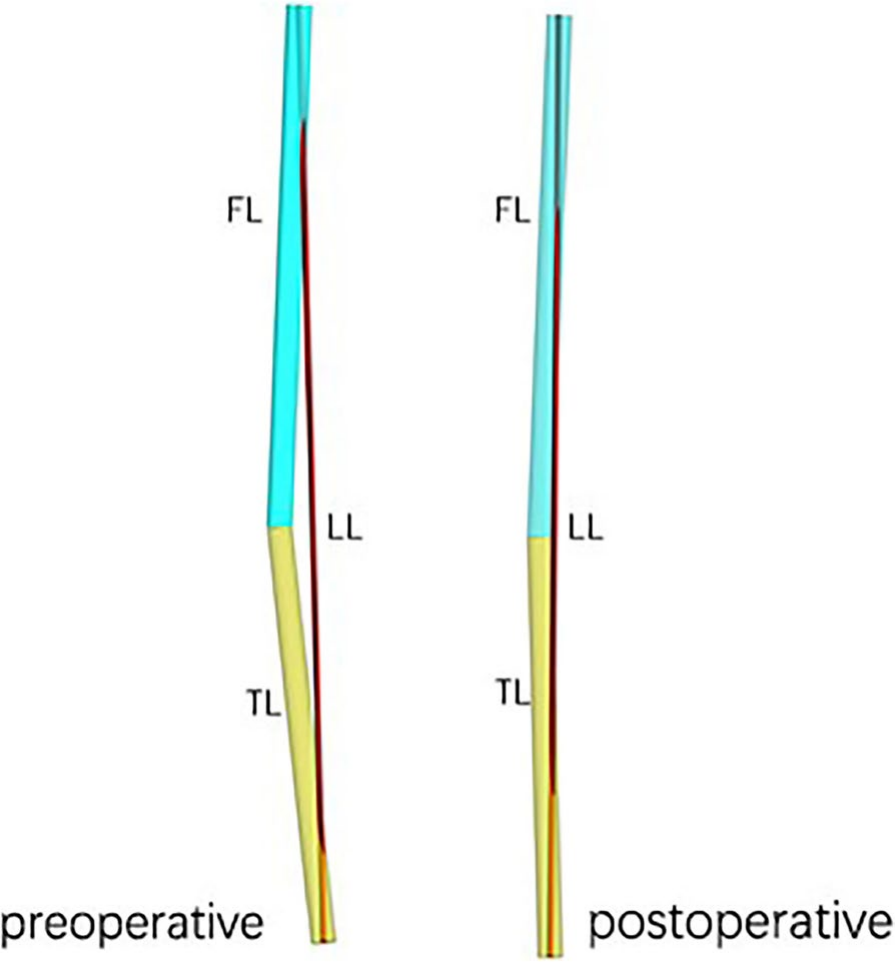
MAYA physical mode. FL (femur length), TL (tibia length), varus angle and flexion contracture angle before and after operation were all averaged, and then the MAYA physical mode calculated the LL (leg length) automatically

76 (87.4%) patients showed an increase in leg length 3 months after the operation, with a mean increase of 0.32 cm (range from -0.30 to 1.05 cm). There was statistically significant difference in leg length before and after operation (*P* < 0.01, Table 2). The leg length was decreased in 11 (12.6%) patients, with an average decrease of 0.10 cm (range from -0.04 to -0.30 cm). Before the second calibration, 56 patients (64.4%) showed leg length lengthening with an average of 0.51 cm (range from -3.03 to 3.37 cm), 31 (35.6%) patients showed leg length shortening, with an average of 0.46 cm (range from -0.06 to -3.03 cm). The physical model showed that average preoperative and postoperative leg lengths were 71.70 cm and 71.96 cm, respectively, indicating an increase of 0.26 cm (Fig. 5). Only 4 (4.6%) patients reported self-perception of increase in leg length after the operation and all of them belonged to the ≥ 5° varus deformity group (Table 1).

**Table 2:** Measurements before and after operation

	Preoperative measurement	Postoperative measurement	Mean difference	*P* value
**Leg Length after first calibration (cm)**	70.65 ± 4.88	71.16 ± 4.92	0.51 ± 0.42	0.063
**Leg Length after second calibration (cm)**	72.36 ± 4.90	72.68 ± 4.86	0.32 ± 0.27	< 0.01
**Varus angle (°)**	8.1 ± 3.7	3.6 ± 2.8	4.6 ± 4.6	< 0.01
**Flexion contracture angle (°)**	8.2 ± 5.5	5.1 ± 4.2	3.1 ± 6.7	< 0.01
**Oxford Knee Score**	23.3 ± 6.3	36.6 ± 4.7	13.3 ± 6.5	< 0.01

**Fig. 6:**
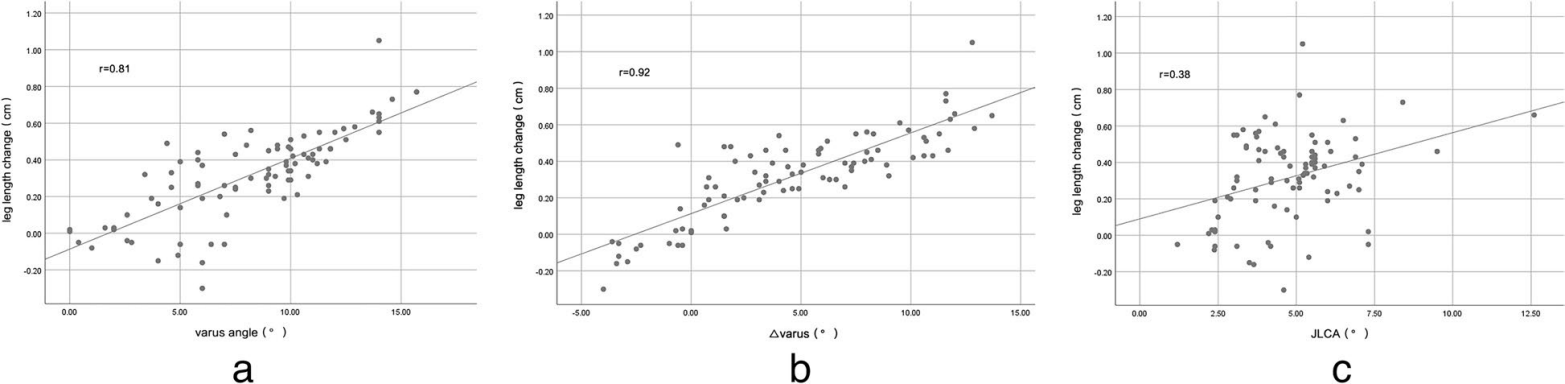
A scatter diagram of the relationship between leg length change and other parameters. **a** varus angle & leg length change (*P* < 0.01, r = 081). **b** △varus & leg length change (*P* < 0.01,r = 0.92). **c** JLCA & leg length change (P = 0.38,r = 0.38)

The mean varus angles preoperatively and postoperatively were 8.1° and 3.6°, respectively. There was a strong correlation between the preoperative varus angle (r = 0.81, *P* < 0.01, Table 3) and varus improvement value (r = 0.92, *P* < 0.01, Fig. 6) with leg length change. The leg length change in the ≥ 5° varus angle group (66 knees) was significantly greater than that in the < 5° varus angle group (*P* < 0.01). The leg length change in the ≥ 5° JLCA group was significantly higher than that in the < 5° JLCA group (*P* < 0.01, Table 4). The mean preoperative and postoperative flexion contractures were 8.2° and 5.1°, respectively. There was no correlation between preoperative flexion contracture and leg length change (*P* = 0.97).

**Table 3:** Correlation of leg length change with other parameters

Parameters	r	*p*-value
**varus angle**	0.81	0.01
**△Varus angle**	0.92	0.01
**JLCA**	0.38	0.01
**FC**	0.04	0.97
**△ FC**	0.11	0.84
**△OKS**	0.03	0.97

*FC* flexion contracture, *JLCA* joint line convergence angle, *OKS* Oxford Knee Score

**Table 4:** Comparison of leg length change between different subgroups

Subgroups		Leg length change (cm)	*p*-value
**varus angle**	≥ 5° (n = 66)	0.39 ± 0.21	0.01
	< 5° (n = 21)	0.09 ± 0.18	
**△Varus angle**	≥ 5° (n = 38)	0.49 ± 0.15	0.01
	< 5° (n = 49)	0.18 ± 0.20	
**JLCA**	≥ 5° (n = 40)	0.39 ± 0.21	0.01
	< 5° (n = 47)	0.25 ± 0.25	

*JLCA* joint line convergence angle

The mean preoperative and postoperative Oxford Knee Scores were 23.3 ± 6.3 and 36.6 ± 4.7, respectively. The Oxford Knee Scores were significantly improved after the operation (mean difference = 14 ± 6.45, *P* < 0.01), and the degree of improvement was not related to the degree of leg length change (*P* = 0.97). There was no statistical difference in Oxford Knee Scores between the those who had a decrease and those who had an increase in leg length change (*P *= 0.99).

## Discussion

The main contribution of this study is that we notice the longitudinal splicing error and eliminate it by using a novel double calibration method. Currently, majority of research have focused on the leg length change after TKA. The most common methodology of examining leg length change is based on the measurement of full-length radiograph with magnification calibration [13–15]. As for MOUKA, to our knowledge, there was only one previous study by Zhao et al. showed that the average lengthening was 9.39 mm (from -21.00 to 33.79 mm) after medial unicompartmental knee arthroplasty [9]. However, we did not expect a significant leg length change in practice, nor should the leg length change have a significant deviation value in that study.

Based on the surgical characteristics of MOUKA, we know that the length of the femur and tibia should remain unchanged before and after the operation with magnification control. Applying this fact, we revised how full-length radiographs are used to measure the length of lower limbs, and added a secondary calibration to eliminate the longitudinal error that might have occurred during the process of splicing. Before the second calibration, the average leg length change in our study was an increase of 0.51 cm (range from -3.03 to 3.37 cm), which was very similar to the results of the previous study and unreasonable.

After double calibrations, we found that 87.4% patients showed an increase of leg length with the average increase of 0.32 cm (range from -0.30 to 1.05 cm). This slight change in length fits the characteristic of MOUKA. Since the femoral prosthesis and tibial prosthesis replaced worn cartilage of approximately equal thickness and the knee joint returns to its normal articular surface after the prosthesis was installed, the leg length change after MOUKA is mainly contributed by the implantation of the bearing on the coronal plane. In our study, the mean bearing thickness was 3.20 mm, equivalent to the mean increase of leg length. Meanwhile, through the physical model, we found that the leg length was extended by 0.26 cm for average, only 0.06 cm difference from our measurements. These all further explains our end results.

In addition, we found that leg length change was strongly correlated with preoperative varus angle and varus improvement value. The leg length change of the ≥ 5° varus angle group was significantly greater than that of < 5° varus angle group (*P* < 0.01). Similarly, the leg length change in the group with ≥ 5° varus angle improvement was significantly greater than that in the group with < 5° varus angle improvement (*P* < 0.01). MOUKA only corrects intraarticular deformities to restore the normal articular surface, so the corresponding imaging data, HKA and HKA improvement value, are strongly correlated with changes in lower extremity length. Our physical model calculated that for every 5° improvement in the varus angle, the lower limb length increased by 0.2 cm. Patients with MOUKA indications had smaller varus deformity before surgery, and MOUKA did not pursue absolute neutral position line of lower limbs, but retained the physiological varus of patients [16]. This led to smaller improvement of the varus angle after the operation, which may be another reason for limited leg length change after MOUKA.

For sagittal deformity, the mean flexion contracture in all patients was 8.2° preoperatively and 5.1° postoperatively. Our statistical analysis did not find a correlation between flexion contracture or improved value with the leg length change. We believe that there are two possible reasons. First, MOUKA does not significantly improve the flexion contracture [17], thus such a small change has little influence on the lower limb length. Second, the flexion contracture is measured from the surface of body rather than from radiograph, with limited accuracy and the possibility of subjective error. Therefore, more precise methods, such as measuring through lateral full-length radiograph, will be needed in the future.

According to 3-month follow-up results, there was no statistically significant difference in Oxford Knee Scores between the group with increased leg length and the group with decreased leg length (*P* = 0.99), indicating that leg length change after MOUKA would not cause short-term functional impact. The study had shown that patients can tolerate the leg length discrepancy of 2 cm [18]. Since the average lengthening of the patients in this group is very slight, we have reason to believe that the length change after MOUKA will not affect the long-term results of patients. In terms of subjective feeling, previous literature shows that most patients could aware the changes in lower limb length greater than 1 cm, and are more sensitive to lower limb lengthening than lower limb shortening [19]. In our study, 83 patients (95.4%) did not perceive leg length change. The other 4 patients perceived lower limb lengthening after operation, with an average of 0.46 cm through radiological measurement. They all belonged to the varus angle ≥ 5° group and varus angle improvement ≥ 5° group, indicating that patients with high degree of varus deformity may perceive such changes.

This study has obvious clinical significance, the double calibration method can produce very precise measurements for leg length change after MOUKA using full-length radiograph. The results were closer to the real situation. It provides a better method for the clinical study of leg length change after MOUKA.

This study also has some limitations: First, this research only studied the leg length change after MOUKA. Further research is required to determine whether this result is applicable to fixed-bearing unicompartmental knee arthroplasty, since it will not overcorrect the lower limb alignment compare to MOUKA [20]. But this slight difference is expected to have limited impact on the findings. In addition, measurement errors may still occur in this novel measurement method proposed, most notably in the measurement of flexion contracture. Therefore, we need to find a more accurate measurement method, for example radiograph measurement, to study the flexion contracture extent for precise influence postoperative leg length change.

## Conclusions

In conclusion, this is the first prospective study to show that the majority of patients experienced a slight increase in leg length after MOUKA. There was a strong correlation between the lengthening and the degree of varus deformity and its improvement. However, the lengthening did not affect the short-term subjective perceptions and short-term function of patients.

## Data Availability

The datasets used and analysed during the current study are available from the corresponding author on reasonable request.

## Abbreviations

MOUKA: Medial mobile-bearing unicompartmental knee arthroplasty
OKS: Oxford knee score
TKA: Total knee arthroplasty
LL: Leg length
FL: Femur length
TL: Tibia length
HKA: Hip-knee-ankle angle
JLCA: Joint line convergence angle
FC: Flexion contracture
